# Factors influencing clinical research participation in ophthalmology: a mixed-methods study in keratoconus

**DOI:** 10.1038/s41598-026-56738-5

**Published:** 2026-06-08

**Authors:** Nikolay Boychev, Kripa Shakya, Jonathan Kopel, Nandini Venkateswaran, Hajirah N. Saeed, Joseph B. Ciolino, Levi N. Kanu

**Affiliations:** 1 Department of Ophthalmology, Harvard Medical School/Massachusetts Eye and Ear/Schepens Eye Research Institute, 20 Staniford Street, Boston, MA 02114 USA; 2 Department of Ophthalmology and Visual Sciences, University of Illinois, Chicago, Chicago, IL USA; 3 Department of Ophthalmology, Loyola University Medical Center, Maywood, IL USA

**Keywords:** Diseases, Health care, Medical research

## Abstract

Keratoconus (KC) disproportionately affects young and underserved populations, yet clinical research participation remains unevenly distributed. To inform equitable recruitment strategies, we investigated demographic, behavioral, and perceptual factors influencing engagement in a non-interventional KC study. We compared demographic and socioeconomic profiles of patients solicited for participation in a non-interventional clinical study involving the laboratory analysis of excess tissue produced during a corneal cross-linking procedure. We conducted structured telephone surveys of participants and non-participants to assess barriers, facilitators, and attitudes toward clinical research. Here we show that, compared to participants, non-participants of the non-interventional KC study were older (33.2 ± 10.3 vs. 27.6 ± 7.3, *p* = 0.001) and predominantly male (90.2% vs. 64.8%, *p* = 0.003), while socioeconomic indices did not differ significantly. Prior clinical research participation strongly predicted future willingness to participate (*p* = 0.01). Key barriers included concerns about side effects, insurance copays, and job flexibility. Facilitators included transportation assistance, provider-led information sessions, and work absence documentation. Despite participants and non-participants sharing similar preferred language profiles, non-participants were significantly more likely to report availability of translated materials as important interventions (*p* = 0.04). Demographic variables alone did not predict participation. Behavioral and contextual factors, rather than fixed demographics, shaped clinical research engagement. Strategies such as personalized provider outreach, logistical support, and culturally tailored materials may improve recruitment and retention in KC clinical research; supporting participant-centered frameworks that address practical needs and readiness to engage, particularly in underserved populations.

**Supplementary Information:**

The online version contains supplementary material available at 10.1038/s41598-026-56738-5.

## Introduction

Keratoconus (KC) is a progressive corneal ectasia marked by stromal thinning, conical protrusion, and irregular astigmatism that primarily affects people in their late teens to early 40s, and if left untreated, can culminate in corneal scarring, acute hydrops, and other vision-threatening complications that may impact quality of life^1^. Corneal crosslinking (CXL) has transformed KC management as the only intervention capable of halting disease progression by strengthening corneal biomechanics^2^. Yet, insurance coverage gaps, socioeconomic constraints, and geographic disparities continue to delay the timely provision of CXL^3–5^. Moreover, minority and disadvantaged populations remain underrepresented in KC research, jeopardizing both external validity and equitable therapeutic advancement^6^.

Despite longstanding Food and Drug Administration and National Institutes of Health mandates to bolster diversity in clinical trials, participation by Black, Hispanic, and other underrepresented groups remains disproportionately low^7^. In 2020, out of approximately 32,000 participants in United States (US) drug trials, only 8% of enrollees were Black, 11% Hispanic, and 6% Asian, even as these populations shoulder a higher burden of chronic disease^8^. In KC specifically, Black and Hispanic patients are more frequently diagnosed with advanced disease, while less likely to receive CXL^9,10^, more prone to acute hydrops^11–13^, more often require keratoplasty^14^, and more often have poorer post-keratoplasty outcomes^15^. Contributing factors include limited insurance coverage, low health literacy, work-related constraints, income, education, geography, and recruitment materials that lack cultural or linguistic congruence^11–13,16,17^. Moreover, our preliminary data indicate that KC patients with Down syndrome and those covered by public health insurance plans face a markedly higher risk of acute hydrops, underscoring the human cost of untreated KC^18^.

Effective clinical trial enrollment hinges on addressing both logistical and perceptual barriers. Transportation difficulties, scheduling conflicts, work commitments, mistrust, and insufficient understanding of research aims have all been cited as major barriers to appointment attendance and health care^19–24^. Prior participation experience in KC clinical research studies itself appears to foster ongoing engagement, suggesting that early inclusion may improve trust and clinical trial literacy^16,17^. However, the relative influence of prior participation versus modifiable supports such as transportation assistance, work-absence documentation, tailored education, and culturally sensitive recruitment materials has not been evaluated in the KC population. Such influences may skew a population that is typically younger and spans a wide socioeconomic range due to the early age of KC onset and the varied barriers patients face in accessing specialty care compared to the general public.

While clinical research enrollment disparities are common, the specific underlying factors leading to these disparities may be case-dependent and still require further elucidation. Without understanding these factors, disparities in enrollment will persist and may translate to worsened disparities in disease understanding, severity, and outcomes. To address these gaps, we conducted a mixed-method survey among KC patients who underwent CXL to compare:^1^ willingness to engage in KC research;^2^ demographic and socioeconomic profiles of clinical study participants versus non-participants; and^3^ identify barriers and facilitators to clinical research enrollment between survey respondents and non-respondents. By elucidating these drivers of participation, we aim to equip eye care providers, clinical trial designers, and institutional stakeholders with actionable guidance for designing more patient-centered recruitment frameworks; thereby ensuring that emerging therapies for KC benefit all segments of the affected population.

## Results

### Demographic and socioeconomic differences between participants and non-participants in a clinical study of KC

Of 112 patients undergoing CXL who were solicited to participate in the non-interventional KC study, 71 (63.4%) elected to participate (i.e., participants), while 41 (36.6%) declined (i.e., non-participants). Non-participants were significantly older than participants (33.2 ± 10.3 vs. 27.6 ± 7.3 years; *p* = 0.001) and demonstrated a greater male predominance (90.2% vs. 64.8%; *p* = 0.003), Table 1.

**Table 1:** Demographic and socioeconomic characteristics of study candidates in a clinical study of keratoconus.

	Participants (*n* = 71)	Non-Participants (*n* = 41)	Total (*n* = 112)	*P* Value
Age (years), mean (SD)	27.6 (7.3)	33.2 (10.3)	29.6 (8.9)	0.001
**Age (years)**,** N (%)**				
**< 20**	10 (14.1)	3 (7.3)	13 (11.6)	0.37
**20–29**	39 (54.9)	11 (26.8)	50 (44.6)	**0.006**
**30–39**	18 (25.4)	18 (43.9)	36 (32.1)	0.06
**> 39**	4 (5.6)	9 (22.0)	13 (11.6)	**0.01**
**Sex (Male)**,** N (%)**	46 (64.8)	37 (90.2)	83 (41.1)	**0.003**
**Race (White)**,** N (%)**	35 (49.3)	26 (63.4)	61 (54.5)	0.15
**Ethnicity (Hispanic)**	14 (19.7)	3 (7.3)	17 (15.2)	0.10
**Preferred Language (English)**,** N (%)**	65 (91.6)	35 (85.4)	100 (89.3)	0.35
**Area Deprivation Index**,** mean (SD)**	32.8 (31.8)	32.5 (30.5)	32.7 (31.2)	0.64
**Primary Insurance (Private)**,** N(%)**	49 (69.0)	30 (73.2)	79 (70.5)	0.67
**Social Vulnerability Index**,** mean (SD)**				
**Overall**	0.44 (0.23)	0.46 (0.21)	0.45 (0.22)	0.27
**Household Characteristics**	0.29 (0.25)	0.35 (0.25)	0.31 (0.25)	0.19
**Racial/Ethnic Status**	0.66 (0.16)	0.64 (0.15)	0.65 (0.16)	0.27
**Housing/Transportation**	0.73 (0.21)	0.74 (0.17)	0.73 (0.20)	0.33
**Socioeconomic**	0.32 (0.22)	0.33 (0.20)	0.32 (0.21)	0.55

Participants (study candidates who opted in to the study) are compared to non-participants (those who opted out).

Socioeconomic place of residence characteristics like Social Vulnerability Index and Area Deprivation Index did not significantly vary between the groups.

### Survey response rates and demographics of respondents

We successfully reached 57 (50.9%) study candidates by telephone and invited their survey responses, Table 2. The study subjects we were able to reach by telephone were more likely to be participants in the CXL study compared to non-participants (59.2% vs. 36.6%, respectively, *p* = 0.02). Moreover, of those subjects reached by telephone, participants were more likely to agree to complete the survey compared to non-participants (95.2% vs. 73.3%, respectively, *p* = 0.04).

**Table 2:** Outreach success and survey completion rates.

Variable	Participants (*n* = 71)	Non-Participants (*n* = 41)	Total (*n* = 112)	*P* value
Telephone Responses				**0.02**
*Reached*	42 (59.2)	15 (36.6)	57 (50.9)	
*Not Reached*	29 (40.8)	26 (63.4)	55 (49.1)	
Survey Responses				**0.04**
*Completed*	40 (95.2)	11 (73.3)	51 (89.5)	
*Declined*	2 (4.8)	4 (26.7)	6 (10.5)	

Participants are patients with unstable keratoconus undergoing CXL who opted in to clinical study participation, whereas non-participants are patients who opted out of participation.

Because the non-participant survey subgroup was small (*n* = 11), all subgroup comparisons should be interpreted as exploratory and descriptive rather than inferential. Consistent with the demographics of our KC population at Mass Eye and Ear (MEE)^11–13,18^, most surveyed study candidates were born in the US, identified as Christian or Agnostic, and had received a college degree, Table 3.

**Table 3:** Demographic and socioeconomic survey results from study candidates in a clinical study of keratoconus.

Survey Respondents	Participants (*n* = 40)	Non-Participants (*n* = 11)	Total (*n* = 51)	*P* value
Age, Mean (SD)	31.7 (8.8)	34.7 (12.7)	32.3 (9.8)	0.36
Sex, N (%)				0.25
*Male*	28 (70.0)	10 (90.9)	38 (74.5)	
*Female*	12 (30.0)	1 (9.1)	13 (25.5)	
Race, N (%)				0.49
*White*	23 (57.5)	8 (25.8)	31 (60.8)	
*Non-White*	17 (42.5)	3 (15.0)	20 (39.2)	
Ethnicity, N (%)				0.99
*Hispanic*	9 (22.5)	2 (18.2)	11 (21.6)	
*Non-Hispanic*	31 (77.5)	9 (81.8)	40 (78.4)	
Civil partnership, N (%)				**0.03**
*Single*	10 (25.0)	7 (63.6)	17 (33.3)	
*Married*	30 (75.0)	4 (36.4)	34 (66.7)	
Religion, N (%)				0.74
*Christian*	25 (62.5)	6 (54.5)	31 (60.8)	
*Non-Christian*	3 (7.5)	2 (18.2)	5 (9.8)	
*Atheist*	3 (7.5)	1 (9.1)	4 (7.8)	
*Agnostic*	9 (22.5)	2 (18.2)	11 (21.6)	
English as native language, N (%)				0.22
*Yes*	33 (82.5)	7 (63.6)	40 (78.4)	
*No*	7 (17.5)	4 (36.4)	11 (21.6)	
Spoken language besides English, N (%)				0.97
*Spanish*	12 (54.5)	2 (50)	14 (53.8)	
*Other*	10 (45.5)	2 (50)	12 (46.2)	
U.S. as place of birth, N (%)				0.72
*Yes*	27 (71.0)	7 (63.6)	34 (69.4)	
*No*	11 (29.0)	4 (36.4)	15 (30.6)	
If “*No*”, at what age did you immigrate to the U.S.? Mean (SD)	16.5 (10.9)	27.3 (4.5)		**0.049**
Education, N (%)				0.78
*(+/-) High School Diploma/(-) College*	13 (32.5)	4 (36.4)	17 (33.3)	
*Completed College*	19 (47.5)	4 (36.4)	23 (45.1)	
*Masters/Doctoral/Professional Degree*	8 (20)	3 (27.3)	11 (21.6)	
Employment, N (%) *Yes* *No*	32 (82.0) 7 (18.0)	8 (72.7) 3 (27.3)	40 (80.0) 10 (20.0)	0.67
Annual Income, N (%)				0.30
*<$50k*	7 (26.9)	1 (16.7)	8 (25)	
*$50-100k*	11(46.2)	1 (16.7)	13 (40.6)	
*$100-150k*	2 (7.7)	2 (33.3)	4 (12.5)	
*> 150k*	5 (19.2)	2 (33.3)	7 (21.9)	

Participants (study candidates who opted in to the study) are compared to non-participants (those who opted out).

No statistically significant differences in age, sex, race, and ethnicity were seen between the participants and non-participants who engaged in our survey. However, surveyed participants were significantly more likely to be married compared to non-participants (75.0% vs. 36.4%; *p* = 0.03). While the proportion of US-born and foreign-born subjects was not statistically different between participant and non-participant groups, foreign-born participants had immigrated to the US at a younger age than foreign-born nonparticipants (16.5 ± 10.9 years vs. 27.3 ± 4.5 years, *p* = 0.049).

### Patient perspectives on clinical research activities

The survey respondents’ general impressions of clinical research and engagement were explored, Table 4.

**Table 4:** Perspectives on clinical research activities at Mass Eye and Ear.

Variable	Participants (*n* = 40)	Non-Participants (*n* = 11)	Total (*n* = 51)	*P* Value
Clinical trial awareness, N (%)				0.28
*Yes*	14 (35)	2 (18.2)	16 (31.4)	
*No*	26 (65)	9 (81.8)	35 (68.6)	
Source of information on clinical trials, N (%)				0.90
*Provider*	9 (64.3)	2 (100)	11 (68.8)	
*Other*	5 (35.7)	0	5 (31.2)	
Patient engagement, N (%)				0.62
*Very poor*	0	0	0	
*Somewhat poor*	0	0	0	
*Neutral*	8 (50)	1 (100)	9 (52.9)	
*Well*	4 (25)	0	4 (23.5)	
*Very well*	4 (25)	0	4 (23.5)	

Participants are patients with unstable keratoconus undergoing CXL who opted in to clinical study participation, whereas non-participants are patients who opted out of participation.

Most (68.6%) survey respondents reported being generally unaware of current clinical research opportunities offered at MEE. Among those who were aware of MEE clinical research activities, most (68.8%) reported their eye care provider as the source of information on such research, and all patients reported either neutral (52.9%) or positive (47.1%) overall rating of MEE patient engagement efforts.

### Perceived barriers to clinical research participation

Surveyed participants were more likely to report being willing to engage in future clinical research compared to non-participants. Specifically, a plurality (47.5%) of participants expressed interest in consistently participating in future research, while only 7.5% reported that they would not likely participate in future research. In contrast no (0%) non-participants expressed a consistent willingness to participate in clinical research, and the majority reported only a conditional interest (72.7%). These differences were statistically significant (*p* = 0.01).

**Fig. 1:**
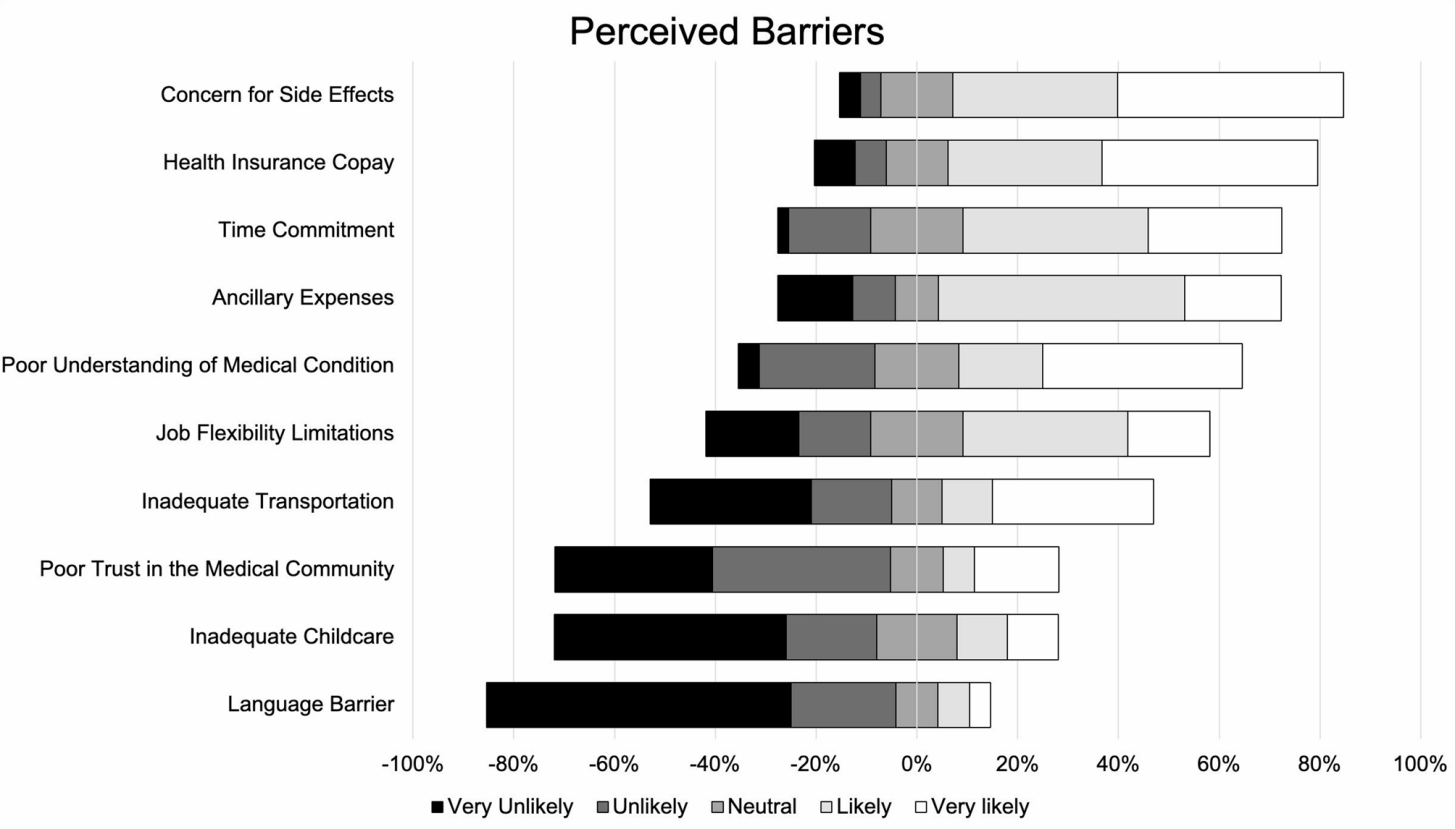
Perspectives on barriers to clinical research participation from survey study candidates on keratoconus. Participants are patients with unstable keratoconus undergoing CXL who opted in to clinical study participation, whereas non-participants are patients who opted out of participation.

For those patients who expressed a conditional interest in future study participation, we solicited input on factors they felt might influence that decision, Fig. 1, Figure S1-S2; Supplementary Table 1.

Common responses included concerns about time commitment, the perceived benefit of the trial, potential side effects, and compensation, which were shared among both participants and non-participants. Additionally, participants suggested that job flexibility, location of the study, and need for additional information on the study would influence their future study participation. In contrast, non-participants suggested that a lack of prior experience participating in clinical studies would be an important factor.

All patients were then asked to rate whether specific common reported barriers to research participation would influence their decision to participate in clinical research.

Among the barriers most frequently reported to influence patients were concerns for side effects of any involved interventions (77.5% *likely* or *very likely* to influence) and concerns for out-of-pocket expenses, including insurance copays and fees (73.4% and 68.1% *likely* or *very likely* to influence, respectively).

The patient’s understanding of their medical condition (i.e., medical condition literacy) was more likely to influence past participants than non-participants (64.1% vs. 22.2% *likely* or *very likely* to influence, *p* = 0.01). In contrast, job flexibility more commonly influenced non-participants than participants (80.0% vs. 41.0% *likely* or *very likely* to influence, respectively, *p* = 0.04). High insurance copay was reported to influence non-participants in all cases (100.0%), compared to 66.7% of participants (*p* = 0.045). Language barrier was more commonly reported as a concern for non-participants (30.0%) than participants (5.2%), a difference which approached significance (*p* = 0.05). The remainder of queried barriers shared similar responses between participants and non-participants (*p* > 0.05).

Most patients (70%) reported that their decision to participate would be influenced by which care team member solicited their participation. Most patients (75.5%) preferred being solicited by their physician, and not another staff member, while the remainder (24.5%) reported no preference. These responses did not differ significantly between participants and non-participants (*p* = 0.30).

### Facilitators to clinical research participation

**Fig. 2:**
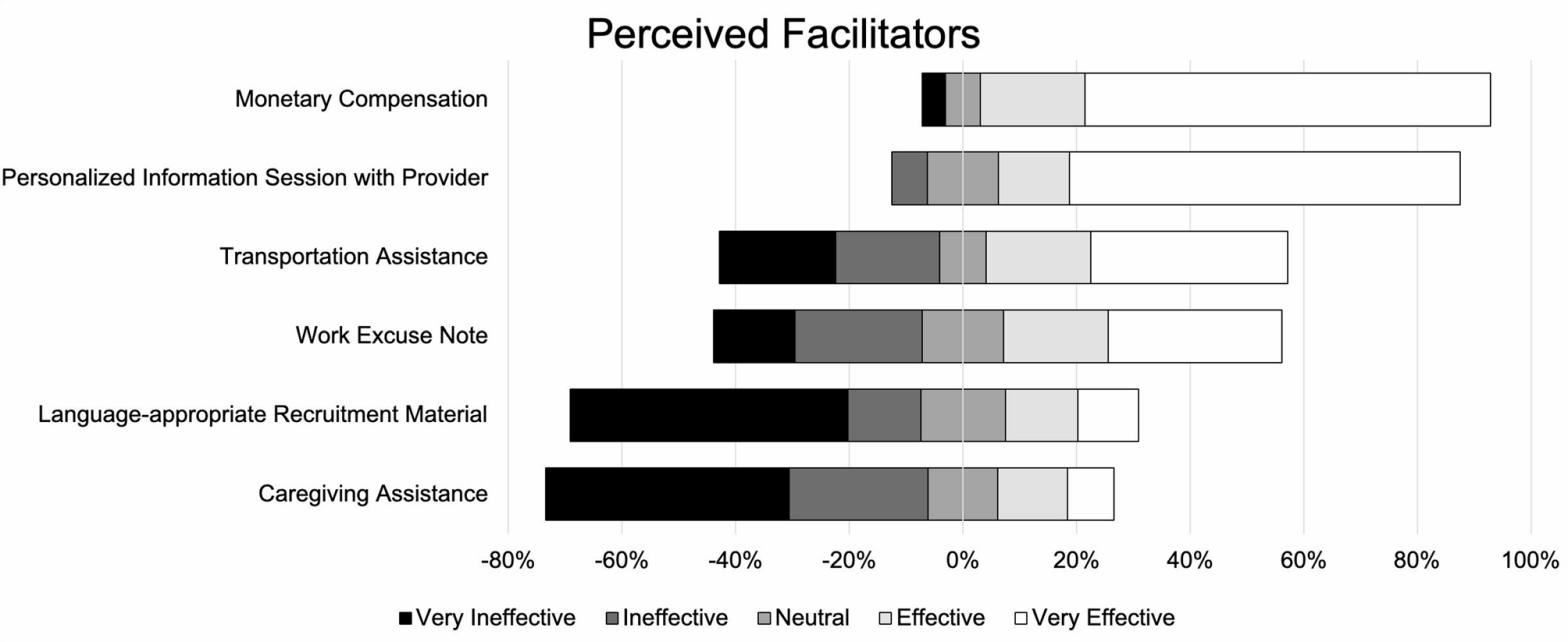
Perspectives on facilitators to clinical research participation from survey study candidates on keratoconus. Participants are patients with unstable keratoconus undergoing CXL who opted in to clinical study participation, whereas non-participants are patients who opted out of participation.

We queried various reported categories of strategies that have been implemented to improve patient participation in clinical research, Fig. 2; Supplementary Table 2.

Overall, reimbursement (89.8%) and provider-led information sessions (81.3%) were the most expected to facilitate respondents’ participation in clinical research. When comparing participants and non-participants, having recruitment material available in the patient’s native language was more likely to be effective in non-participants than participants (50.0% vs. 16.2% *effective* or *very effective* strategy, respectively, *p* = 0.04). Other queried strategies received similar responses between participants and non-participants (*p* > 0.05).

Patients were also queried for strategies that may improve research participation that were not listed. Participants recommended aligning clinical trial visits with existing doctor’s appointments and using online questionnaires for recruitment. Non-participants, on the other hand, suggested providing additional, non-financial incentives, such as offering refreshments.

## Discussion

This study offers critical insight into the factors influencing patient participation in clinical research on KC. An incomplete cross section of the KC patient population, because of skewed participation, may result in an incomplete characterization of disease pathogenesis and could result in a widening gap in patient outcomes. By investigating a comprehensive set of barriers to and facilitators of research participation, we aimed to offer new insights for improving population-specific recruitment and retention strategies and inform large prospective clinical research studies.

One of the most notable findings is the role of prior research experience. Individuals who had previously participated in clinical studies were significantly more likely to express willingness to participate again. This finding agrees with the literature^25^, suggesting that prior clinical research involvement is positively associated with reduced apprehension and greater trust surrounding research activities, which leads to improved engagement. Several logistical and perceptual barriers emerged as important determinants of clinical research enrolment and retention. For instance, participants who rated transportation assistance as “very effective” were much more likely to have previously enrolled in clinical trials. This aligns with past evidence from a multicenter study of 17 research projects at 8 institutions that logistical barriers such as transportation are among the most cited reasons for low research participation among minority and underserved populations in the US^26^. Similarly, providing a doctor’s note to excuse absence from work was associated with significantly increased likelihood of participation. This highlights the importance of facilitating flexibility around work obligations, a modifiable barrier that has received comparatively less attention in the clinical trial recruitment literature. Interestingly, non-participants were significantly more likely to report having recruitment materials available in a patient’s native language as important compared to non-participants despite the two groups not exhibiting a significantly different preferred language profile. The perceived sentiments behind offering recruitment material in languages other than English may be an important issue influencing potential study subjects of different backgrounds, even among native English speakers. Moreover, the translation of study materials should be performed in such a way as to take into account cultural relevance and nuance^27^.

Common demographic variables including age, sex, race and ethnicity, civil partnership, religion, native and preferred language, birthplace, education, employment, or salary were not significantly associated with clinical trial participation. These findings highlight that demographic characteristics alone may not be the primary determinants of research engagement. For instance, a large systematic review of 44 publications spanning the decade between 2000 and 2011 similarly reported that experienced or perceived barriers and facilitators in health research participation, particularly among major US racial/ethnic minorities, are less influenced by such fixed characteristics^28^. Personalized provider-led information sessions, though not statistically significant, were rated as “very effective” by most participants. This suggests a promising, underutilized strategy for improving research engagement, particularly among those unfamiliar with clinical research. Perhaps surprisingly, caregiving assistance and reimbursement did not have a significant effect on research participation. The limited impact of caregiving assistance may be due to a mismatch between perceived and actual caregiving needs in the surveyed population. Meanwhile, although reimbursement was rated as “very effective” by many respondents, its lack of predictive value for participation aligns with previous studies, implying that monetary incentives alone are insufficient to overcome trust or logistical barriers; instead, such motivators for research enrollment should be part of a broader strategy that addresses the full participant experience^29^. Perceptions of trust in the medical community, fear or concern of side effects, and lack of understanding or literacy of one’s medical condition were also not significant predictors of participation, though they remain commonly cited barriers in other studies. This discrepancy may be due to possible ceiling effects, for instance, where the overall trust levels are already high or because of a limited size and specificity of the surveyed population. Our findings suggest that low condition literacy and vague expectations may still discourage engagement among research-naïve patients, highlighting an opportunity for targeted educational outreach. Moreover, while some patients benefit educationally from research participation, others may leave with lingering confusion or unmet informational needs.

Although patient awareness of clinical research opportunities was widespread among respondents, it did not correlate strongly with participation. This included some individuals who had previously participated in a KC study, indicating that their involvement may have been perceived as isolated or disconnected from the institution’s larger research mission. Our finding suggests room for improvement in how research opportunities are communicated and framed to patients. Specifically, enhancing the perceived relevance of a clinical trial to the patient’s medical condition, building trust through personalized interactions, and reducing friction points such as scheduling conflicts and transportation may prove more effective. These findings support a shift away from generalized demographic targeting toward contextual and behavioral strategies designed based on practical needs and readiness to engage in clinical research.

This study has several limitations. The sample size was limited by the number of KC patients who underwent CXL and by the unpredictability of engagement in telephone surveys. Interpretation of subgroup differences is limited by the small number of surveyed non-participants, and these findings should be viewed as hypothesis-generating. Additionally, because this study investigated patient engagement in clinical research (e.g., patients undergoing CXL at a tertiary academic center), it is subject to inherent selection bias (e.g., we reached 50.9% through telephone calls with higher survey response rates among participants) in that the survey respondents may not accurately represent the broader KC population (e.g., underserved populations receive less CXL). Individuals who reach CXL evaluation typically have greater access to specialty care, higher health literacy, and, in our cohort, a disproportionately high level of educational attainment. These characteristics may influence both their awareness of clinical research and their perceived barriers to participation. Patients who have not yet accessed specialty care, who face greater socioeconomic barriers, or who are managed in community settings may experience different challenges and motivations. Because disease severity metrics such as best-corrected visual acuity or tomographic indices were not collected, we were unable to assess whether severity correlated with willingness to participate. Future work should include patients across the full spectrum of care settings and disease severity. Future studies may also improve representativeness by administering surveys at the time of clinical encounter or immediately following the recruitment discussion, which could improve non-response bias and capture a broader range of patient perspectives. Hence, the study’s non-interventional design and lack of follow-up visits potentially shaped the observed profile, which may limit the generalizability to interventional studies where sustained engagement and procedural demands may alter patient recruitment strategies. While our survey included open-ended questions that were designed to elicit diverse perspectives, the format still lacked the capacity to capture granular details of patient sentiments. Despite efforts to optimize question framing and encourage elaboration, responses remained limited in contextual nuance. More subjective, conversational settings such as interviews and focus groups will be essential to uncover the motivations and deterrents that quantitative approaches may overlook. These efforts will be crucial to advancing effective engagement strategies in clinical research, particularly in diseases like KC that often affect young and underserved populations^11–13,18^. Finally, the survey did not assess general attitudes toward science or biomedical research, and we were unable to determine whether such attitudes influenced willingness to participate. Future work incorporating validated measures of research trust and scientific orientation will be important to understand these potential effects.

In summary, this study reinforces that prior clinical research experience is a strong facilitator of future participation; perceived barriers (e.g., side effects, expenses, time commitment, insurance, job flexibility) and facilitators (e.g., reimbursement, personalized information session with the provider, transportation assistance, and accommodations for work obligations) play a significant role in encouraging participation and should be integrated into future recruitment frameworks; and educational outreach that clarifies the purpose, benefits, and processes of clinical research, especially among individuals with limited literacy of their medical condition, holds promise for improving enrollment. To translate these findings into practice, we recommend: investing in structured transportation support as part of clinical trial infrastructure; offering institutionally supported work absence documentation; and developing culturally and contextually tailored recruitment materials in collaboration with community stakeholders. Recruitment strategies may also benefit from offering patient information in more digestible formats, such as brief leaflets or videos with opportunities for real-time questions, which could improve comprehension and indirectly support higher participation rates. Additionally, expanding personalized educational interventions led by providers may build trust and engagement, especially for those without prior research exposure.

## Methods

### Sex as a biological variable

Both male and female participants were included in the analyses. Sex was not the primary variable of interest and was not used to define subgroups. No exclusion criteria were based on sex.

### Statistics

We compared participants to non-participants using χ² tests or Fisher’s exact tests for categorical variables and Kruskal-Wallis tests for continuous variables. Normality of continuous variables was assessed using the Shapiro-Wilk test. Survey responses were analyzed only if complete; Likert-type items were treated as ordinal scales. Statistical significance was set at α = 0.05. Data were de-identified and stratified sampling applied to ensure representativeness.

### Study approval

This clinical study and survey were approved by the Institutional Review Board (protocol 2024P001953) at MEE. Written informed consent was received prior to participation. Written informed consent was obtained from all participants prior to initiating the telephone survey, in accordance with the institutional review board-approved protocol for minimal-risk research.

### Data availability

The datasets used and analyzed during the current study are available from the corresponding author on reasonable request.

### Study design

**Fig. 3:**
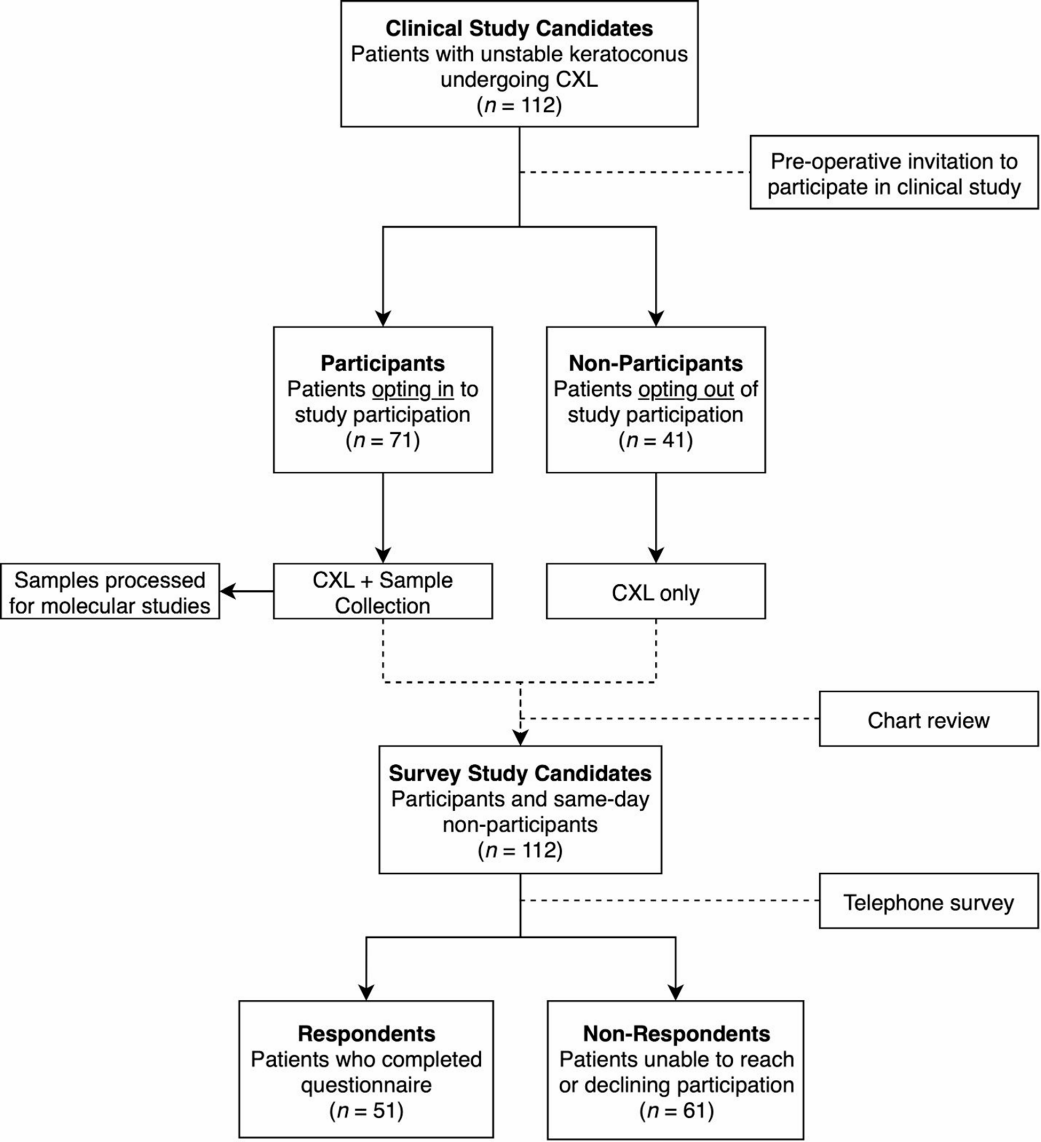
Flowchart of the study design.

We investigated enrollment patterns in KC clinical research and identified factors that influence patient enrollment in these studies to ultimately guide the implementation of methods to reduce disparities in current and future clinical research study enrollment. We conducted a sequential, mixed-method study involving patients who were approached for a non-interventional, no-more-than-minimal-risk observational study requiring no additional procedures and participated in clinical research in the Cornea Service at MEE (Fig. 3).

Clinical study candidates were identified by the treating provider and included all adults (≥ 18 years old) with a clinical diagnosis of unstable KC who underwent CXL at MEE between January 2020 and December 2023. Exclusion criteria included evidence of infectious diseases, pregnancy, and prior ocular surgery. This clinical study involved the collection of corneal epithelium for laboratory analysis following their CXL procedure and did not involve any interventions or follow-up study visits. For patients who elected to participate in the clinical study, corneal tissue collection consisted solely of the standard central epithelial debridement routinely performed at the start of epithelium-off CXL. No additional epithelial removal or other procedures were performed. Participants were explicitly informed that the procedure would not differ from standard clinical care and that the only study-related activity was the laboratory analysis of tissue that would otherwise be discarded. Participation in the study did not substantively alter the duration, location, or workflow of the CXL procedure. No additional time, travel, or procedural steps were required. No compensation, reimbursement, or other incentives (e.g., paid parking) were offered.

Once study enrollment was complete, we completed a retrospective cohort analysis of study candidates. Those who had participated in this clinical study (participants) were compared to those who elected not to participate (non-participants). Basic demographic data (e.g., age, sex, and ethnicity) from electronic medical records were collected and compared between study participants and non-participants. Socioeconomic characteristics of a subject’s place of residence were obtained from the documented home address, using publicly available repositories^30–32^.

Subsequently, we conducted a telephone survey of all study-eligible patients (participants and non-participants) to understand the factors influencing their decisions. The Exclusion criteria included a lack of consent to be contacted for research.

This research was conducted according to the tenets of the Declaration of Helsinki and in compliance with the Health Insurance Portability and Accountability Act^33^.

### Questionnaire development and implementation

We performed a focused literature review of enrollment-based clinical research studies using PubMed and Google Scholar to compile a weighted list of known barriers and facilitators to study participation^34–42^. Searches included combinations of the following MeSH terms and keywords: ‘keratoconus,’ ‘clinical research participation,’ ‘clinical trial recruitment,’ ‘barriers,’ ‘facilitators,’ ‘health literacy,’ ‘transportation,’ ‘socioeconomic factors,’ and ‘underrepresented populations.’ Articles published in English between 1995 and 2024 were considered. We prioritized systematic reviews, multicenter studies, and empirical research examining barriers and facilitators to clinical trial participation in ophthalmology and other chronic diseases. Reference lists of key publications were also screened to identify additional relevant studies. The factors that were used to inform both the data-extraction fields and survey item pool were divided into the following categories: sociodemographic (e.g., age, sex, race/ethnicity, insurance status, employment, zip code), clinical variables (e.g., comorbidities, body mass index (BMI)), and clinical scheduling data (e.g., total visits and missed appointments).

We generated a 25-item survey covering logistical (e.g., transportation, scheduling), perceptual (e.g., trust, literacy), and cultural (e.g., language, place of birth) domains. The full survey instrument, including exact question wording and response options, is provided in Supplementary Document 1. The survey was pilot-tested with five KC patients to assess clarity, flow, and comprehension; minor wording adjustments were made prior to deployment. Telephone surveys required approximately 8–12 min to complete. There were two incomplete surveys, which were excluded because missing responses prevented consistent comparison across items; however, the proportion of incomplete surveys was small and did not materially affect the distribution of respondent characteristics. Surveys were administered by trained clinical staff via telephone; interpreter services were used for non-English speakers.

## Supplementary Information

Below is the link to the electronic supplementary material.

Supplementary Material 1

Supplementary Material 2

## Data Availability

The datasets used and analyzed during the current study are available from the corresponding author on reasonable request.

## Abbreviations

KC: Keratoconus
CXL: Corneal collagen crosslinking
MEE: Massachusetts Eye and Ear
BMI: Body mass index
US: United States
