# Supplementary material for: Factors influencing clinical research participation in ophthalmology: a mixed-methods study in keratoconus
Source: Sci Rep. 2026 Jun 8;16:25970. doi: 10.1038/s41598-026-56738-5 (PMC13486715; doi:10.1038/s41598-026-56738-5)
Supplement: Supplementary file 1 [file 41598_2026_56738_MOESM1_ESM.docx]

KCN Enrollment Questionnaires

Enhancing Clinical Trial Participation in Keratoconus: Development and Evaluation of Engagement Strategies for Underrepresented Populations

ALL SUBJECTS

1. Age
2. Address
3. Race [White, Black or African American, Native American or American Indian, Pacific Islander, East Asian, Indian, Middle Eastern/Arabic, Other (Please Specify):]
4. Ethnicity (Hispanic, Not Hispanic)
5. Marital Status (Married, Single, Separated/Divorced)
6. Religion [Christian, Jewish, Muslim, Buddhist, Hindu, Spiritual but not religious, Atheist, Agnostic, Other (Please Specify)]
7. Born in the United States? (Yes, No)
   a. If no, at what age did you immigrate to United States?
   b. If no, would you say you identify closer with American culture or your native culture? (American culture, Native culture)
8. Is English your native language? (Yes, No)
9. If no, would you consider yourself to be fluent in both spoken and written English? (Yes, No)
10. Besides English, what other languages do you speak? [Spanish, Portuguese, Hindi, Mandarin, Other (Please Specify)]
11. From those, which is your preferred language? [Spanish, Portuguese, Hindi, Mandarin, Other (Please Specify)]
12. What is the highest degree of school you have completed? [Less than high school diploma/GED, High school diploma/GED (completed), Trade/technical/vocational training, Some college (not completed), 4-year college (completed), Doctoral/professional degree (completed)]
13. Are you currently employed? (Yes, No)
    a. If yes: [Employed full time/ self -employed (at least 40 hrs/week), Employed part time (less than 40 hrs/week), Student, Other (Please specify)]

If no: [Retired, Homemaker, Disabled, Other (please specify)]

14. What is your annual household income? (Less than $50,000, $50,000 - $100,000, $100,000 - $150,000, More than $150,000)

ALL SUBJECTS

1. In general, have you been made aware of clinical trials at Mass Eye and Ear? (Yes, No)
   a. If yes, who have you heard from? (Provider, Other clinical staff, Advertisement, Friend, Other)
2. How well do you feel patients are engaged to participate? (Very poorly, Somewhat poorly, Neutral, Well, Very well)

Whether or not you have participated in a clinical trial before, we are trying to better understand the motivators behind clinical trial participation.

1. In general, would you ever consider participating in a clinical trial? (Never, It depends, Regularly)
   a. If never, what are your reservations?
   b. If it depends, what does it primarily depend on?

We have different ways of recruiting patients into clinical trials. For example, we might ask you during a clinical visit, or you might find out about it on your own through websites or flyers or a community health center.

1. If you had a choice, would you have a preference for how you would like to be recruited? [Your doctor, Clinic staff (technician/nurse), Flyers in the clinic, Website links, Community health worker, Other (Please specify), No preference]

There are many factors that might make participating in clinical trials difficult - we are going to name a few of them. Please let us know how likely each of the following factors might be to influence your decision to participate.

1. Lack of transportation (Very unlikely/ unlikely/neutral/likely/very likely)
2. Lack of childcare/other caregiving responsibilities (Very unlikely/ unlikely/neutral/likely/very likely)
3. High insurance copay/hospital bills (Very unlikely/ unlikely/neutral/likely/very likely)
4. Limited job flexibility (Very unlikely/ unlikely/neutral/likely/very likely)
5. Time commitment (Very unlikely/ unlikely/neutral/likely/very likely)
6. Impact on income (Very unlikely/ unlikely/neutral/likely/very likely)
7. Language barrier (Very unlikely/ unlikely/neutral/likely/very likely)
8. Knowledge of your condition (Very unlikely/ unlikely/neutral/likely/very likely)
9. Lack of trust in the medical community (Very unlikely/ unlikely/neutral/likely/very likely)
10. Concern about side effects of participating (Very unlikely/ unlikely/neutral/likely/very likely)
11. No reason
12. Please specify if there are any other barriers:

Sometimes, clinical trials are able to provide benefits to patients who participate. We are trying to determine which benefits would be most effective at encouraging participation. We are going to name a few - please let us know how effective each benefit might be for you.

1. Full transportation assistance (Very ineffective/ Ineffective/Neutral/Effective/Very effective)
2. Childcare or caregiving assistance (Very ineffective/ Ineffective/Neutral/Effective/Very effective)
3. Reimbursement for participation (Very ineffective/ Ineffective/Neutral/Effective/Very effective)
4. A doctor's note to excuse you from work (Very ineffective/ Ineffective/Neutral/Effective/Very effective)
5. Recruitment materials that were available in your language (Very ineffective/ Ineffective/Neutral/Effective/Very effective)
6. Personalized information session with a provider (Very ineffective/ Ineffective/Neutral/Effective/Very effective)
7. Other (Please specify):

CLINICAL TRIAL QUESTIONS

1. Have you ever participated in a clinical study or trial (does not have to be MEE)? (Yes, No)
2. If yes, have you ever left a study early (not completed the full study)? (Never, Once, Rarely, Sometimes, Frequently)
3. You may have been asked to participate in a clinical study for XXX on XXX date by XXX provider. Do you recall being engaged about this? (Yes, No)
   a. If yes, do you recall whether you chose to participate? (Yes, No)
   b. If do not recall, provide the study name and what was involved as a reminder.

CONFIRMED PARTICIPANTS

1. How well would you say the study and its procedures were explained? (Very poorly, Poorly, Neutral, Well, Very well)
2. How confident were you in your understanding of what the study entailed, including procedures, benefits, risks, etc.? (Very poorly, Poorly, Neutral, Well, Very well)
3. When you participated in a clinical study, do you feel your knowledge of your condition improved, stayed the same, or worsened? (Improved, Stayed the same, Worsened)
4. Has being involved in this clinical study affected your likelihood of future trial participation? (Less likely, No effect, More likely)
5. How likely are you to recommend that others participate in clinical trials? (Very unlikely/ unlikely/neutral/likely/very likely)

CONFIRMED NON-PARTICIPANTS

1. Do you recall the primary reason for not participating in this specific study? [Lack of childcare / other caregiving responsibilities, Distance from home / transportation difficulty, Language barrier, Insurance coverage, Limited job flexibility, Impact on income, Time commitment, Knowledge of your condition, Lack of trust in the medical community, Concern about the side effects of participating, No reason, Other (Please specify)]
2. Are there any specific changes that would improve your likelihood of participation?
