# Supplementary material for: Factors influencing clinical research participation in ophthalmology: a mixed-methods study in keratoconus
Source: Sci Rep. 2026 Jun 8;16:25970. doi: 10.1038/s41598-026-56738-5 (PMC13486715; doi:10.1038/s41598-026-56738-5)
Supplement: Supplementary file 2 [file 41598_2026_56738_MOESM2_ESM.docx]

**Supplementary Table 1.** **Survey responses: perceived barriers to clinical research participation.**

| **Variable** | **Participants** | **Non-Participants** | **Total** |
| --- | --- | --- | --- |
| Poor Understanding of Medical Condition, N (%)  *Very unlikely*  *Unlikely*  *Neutral*  *Likely*  *Very likely* | 1 (2.6)  6 (15.4)  7 (17.9)  7 (17.9)  18 (46.2) | 1 (11.1)  5 (55.6)  1 (11.1)  1 (11.1)  1 (11.1) | 2 (4.2)  11 (22.9)  8 (16.7)  8 (16.7)  19 (39.6) |
| Job Flexibility Limitations, N (%)  *Very unlikely*  *Unlikely*  *Neutral*  *Likely*  *Very likely* | 8 (20.5)  6 (15.4)  9 (23.1)  9 (23.1)  7 (17.9) | 1 (10)  1 (10)  0  7 (70)  1 (10) | 9 (18.4)  7 (14.3)  9 (18.4)  16 (32.6)  8 (16.3) |
| Health Insurance Copay, N (%)  *Very unlikely*  *Unlikely*  *Neutral*  *Likely*  *Very likely* | 4 (10.3)  3 (7.7)  6 (15.4)  9 (23.1)  17 (43.6) | 0  0  0  6 (60)  4 (40) | 4 (8.2)  3 (6.1)  6 (12.2)  15 (30.6)  21 (42.9) |
| Time Commitment, N (%)  *Very unlikely*  *Unlikely*  *Neutral*  *Likely*  *Very likely* | 1 (2.6)  4 (10.3)  8 (20.5)  14 (35.9)  12 (30.8) | 0  4 (40)  1 (10)  4 (40)  1 (10) | 1 (2)  8 (16.3)  9 (18.4)  18 (36.7)  13 (26.5) |
| Language Barrier, N (%)  *Very unlikely*  *Unlikely*  *Neutral*  *Likely*  *Very likely* | 25 (65.8)  8 (21.1)  3 (7.9)  1 (2.6)  1 (2.6) | 4 (40)  2 (20)  1 (10)  2 (20)  1 (10) | 29 (60.4)  10 (20.8)  4 (8.3)  3 (6.3)  2 (4.2) |
| Ancillary Expenses, N (%)  *Very unlikely*  *Unlikely*  *Neutral*  *Likely*  *Very likely* | 6 (16.2)  3 (8.1)  2 (5.4)  17 (45.9)  9 (24.3) | 1 (10)  1 (10)  2 (20)  6 (60)  0 | 7 (14.9)  4 (8.5)  4 (8.5)  23 (48.9)  9 (19.1) |
| Inadequate Childcare, N (%)  *Very unlikely*  *Unlikely*  *Neutral*  *Likely*  *Very likely* | 21 (52.5)  6 (15)  6 (15)  3 (7.5)  4 (10) | 2 (20)  3 (30)  2 (20)  2 (20)  1 (10) | 23 (46)  9 (18)  8 (16)  5 (10)  5 (10) |
| Inadequate Transportation, N (%)  *Very unlikely*  *Unlikely*  *Neutral*  *Likely*  *Very likely* | 13 (32.5)  7 (17.5)  5 (12.5)  3 (7.5)  12 (30) | 3 (30)  1 (10)  0  2 (20)  4 (40) | 16 (32)  8 (16)  5 (10)  5 (10)  16 (32) |
| Concern for Side Effects, N (%)  *Very unlikely*  *Unlikely*  *Neutral*  *Likely*  *Very likely* | 2 (5.1)  2 (5.1)  6 (15.4)  11 (28.2)  18 (46.2) | 0  0  1 (10)  5 (50)  4 (40) | 2 (4.1)  2 (4.1)  7 (14.3)  16 (32.7)  22 (44.9) |
| Poor Trust in the Medical Community, N (%)  *Very unlikely*  *Unlikely*  *Neutral*  *Likely*  *Very likely* | 11 (28.9)  13 (34.2)  4 (10.5)  3 (7.9)  7 (18.4) | 4 (40)  4 (40)  1 (10)  0  1 (10) | 15 (31.3)  17 (35.4)  5 (10.4)  3 (6.3)  8 (16.7) |
| Decision to Participate in a Clinical Trial may be Influenced by Who is Asking, N (%)  *Yes*  *No*  Preference for Who is Recruiting, N (%)  *Provider*  *Other*  *No preference* | 28 (70)  12 (30)  29 (72.5)  0  11 (27.5) | 7 (70)  3 (30)  8 (88.9)  0  1 (11.1) | 35 (70)  15 (30)  37 (75.5)  0  12 (24.5) |

Subjects were asked to rate how likely each variable would be to influence their decision to participate in clinical research.

**Figure S1**. Perceived barriers of participation in clinical research; KC study participants.

**Figure S2**. Perceived barriers of participation in clinical research; KC study non-participants.

**Supplementary Table 2.** **Survey responses: perceived facilitators to clinical research participation.**

| **Variable** | **Participants** | **Non-Participants** | **Total** |
| --- | --- | --- | --- |
| Transportation Assistance, N (%)  *Very ineffective*  *Ineffective*  *Neutral*  *Effective*  *Very effective* | 8 (20.5)  5 (12.8)  4 (10.3)  5 (12.8)  17 (43.6) | 2 (20)  4 (40)  0  4 (40)  0 | 10 (20.4)  9 (18.4)  4 (8.2)  9 (18.4)  17 (34.7) |
| Work Excuse Note, N (%)  *Very ineffective*  *Ineffective*  *Neutral*  *Effective*  *Very effective* | 5 (12.8)  9 (23.1)  6 (15.4)  4 (10.3)  15 (38.5) | 2 (20)  2 (20)  1 (10)  5 (50)  0 | 7 (14.3)  11 (22.4)  7 (14.3)  9 (18.4)  15 (30.6) |
| Language-appropriate Recruitment Material, N (%)  *Very ineffective*  *Ineffective*  *Neutral*  *Effective*  *Very effective* | 22 (59.5)  5 (13.5)  4 (10.8)  4 (10.8)  2 (5.4) | 1 (10)  1 (10)  3 (30) 2 (20)  3 (30) | 23 (48.9)  6 (12.8)  7 (14.9)  6 (12.8)  5 (10.6) |
| Personalized Information Session with Provider, N (%)  *Very ineffective*  *Ineffective*  *Neutral*  *Effective*  *Very effective* | 0  1 (2.6)  6 (15.4)  4 (10.3)  28 (71.8) | 0  2 (22.2)  0  2 (22.2)  5 (55.6) | 0  3 (6.3)  6 (12.5)  6 (12.5)  33 (68.8) |
| Caregiving Assistance, N (%)  *Very ineffective*  *Ineffective*  *Neutral*  *Effective*  *Very effective* | 19 (48.7)  9 (23.1)  3 (7.7)  5 (12.8)  3 (7.7) | 2 (20)  3 (30)  3 (30)  1 (10)  1 (10) | 21 (42.9)  12 (24.5)  6 (12.2)  6 (12.2)  4 (8.2) |
| Monetary Compensation, N (%)  *Very ineffective*  *Ineffective*  *Neutral*  *Effective*  *Very effective* | 2 (5.1)  0  2 (5.1)  6 (15.4)  29 (74.4) | 0  0  1 (10)  3 (30)  6 (60) | 2 (4.1)  0  3 (6.1)  9 (18.4)  35 (71.4) |

Subjects were asked to rate how likely each variable would be to influence their decision to participate in clinical research.

**Figure S3**. Perceived facilitators of participation in clinical research; KC study participants.

**Figure S4**. Perceived barriers of participation in clinical research; KC study non-participants.
